# Low anterior chamber volume as a risk factor in non-arteritic anterior ischemic optic neuropathy

**DOI:** 10.3389/fopht.2025.1554279

**Published:** 2025-05-20

**Authors:** Durgul Acan, Beyza Betul Cakar, Eyyup Karahan

**Affiliations:** Department of Ophthalmology, Balıkesir University School of Medicine, Balıkesir, Türkiye

**Keywords:** anterior chamber depth, anterior chamber volume, central corneal thickness, iridocorneal angle, nonarteritic anterior ischemic optic neuropathy

## Abstract

**Purpose:**

This study aimed to compare the anterior chamber depth (ACD), anterior chamber volume (ACV), and iridocorneal angle (ICA) of the eyes of patients with non-arteritic anterior ischemic optic neuropathy (NAION) and normal eyes.

**Methods:**

In this cross-sectional study, 28 patients with NAION who were admitted to our institution were examined. Central corneal thickness (CCT), ACV, ACD, and ICA of all eyes were measured using corneal topography (Sirius, CSO, Italy). Axial lengths (ALs) were measured using an IOL-Master 500 (Carl Zeiss, Meditec). The eyes of these patients were compared with the eyes of 29 healthy individuals of similar age and gender.

**Results:**

The mean ALs of the eyes with NAION and those in the control group were not statistically different, measuring 22.95 ± 0.68 mm and 23.13 ± 0.80mm, respectively (p=0.651). While the average ACV was 137.93 ± 41.01 mm^3^ in the control group, it was significantly lower at 117.86 ± 22.23 mm^3^ in the patients with NAION (p=0.038). The mean ACD, ICA, and CCT values in the control and study groups were not statistically different, with 2.82 ± 0.57 mm and 2.64 ± 0.31 mm, 41.62 ± 6.99° and 40.14 ± 7.04°, and 542.48 ± 19.39µm and 544.68 ± 31.26 µm, respectively (p1 = 0.236, p2 = 0.693, and p3 = 0.959). No statistical differences were found between the eyes with NAION and their fellow eyes in terms of AL, CCT, ACD, ACV, and ICA (p>0.05).

**Conclusion:**

Differences in anterior segment morphology were observed in eyes with NAION compared to healthy eyes. Decreased ACV may be a risk factor for NAION.

## Introduction

Non-arteritic ischemic optic neuropathy (NAION) is the most prevalent type of ischemic optic neuropathy in patients over 50 years of age (1, 2). The pathophysiology of NAION has not been fully elucidated yet. However, it is claimed that the crowding of the disc leads to a type of compartment syndrome, inhibiting axonal flow and causing vascular insufficiency related to existing systemic vascular diseases (3–6). The fellow eyes of these patients often also exhibit a small and crowded optic disc, commonly referred to as “disc at risk” in the literature. The presence of “disc at risk” indicates a high risk of developing NAION (7).

Differences in the anterior segment, such as corneal thickness and corneal biomechanics, have been previously determined in some diseases, such as normotensive glaucoma (8, 9). In a previous study conducted at our clinic, it was demonstrated that patients with central retinal vein occlusion (CRVO) exhibit anterior segment crowding, characterized by a reduced anterior chamber volume (ACV), resembling the anatomical configuration observed in a crowded optic nerve head (10). Only corneal thickness and hysteresis were evaluated in some studies on the relationship between NAION and anterior segment morphology (11, 12). The aim of this study was to compare anterior chamber morphology in NAION with normal eyes.

## Materials and methods

In this cross-sectional study, 28 eyes of 28 patients who were followed up and treated with the diagnosis of NAION between May 2019 and January 2023 were included. Furthermore, 29 healthy eyes from 29 age- and gender-matched individuals with comparable axial lengths (ALs) were enrolled as the control group. The ethics committee of the university evaluated and approved this study in compliance with the guidelines outlined in the Declaration of Helsinki. All the patients in this study were informed about the purposes of the study, and written consent was obtained from all participants.

The diagnosis of NAION was described as an acute loss of visual acuity without pain, visual field abnormalities, an ipsilateral relative afferent pupillary deficiency, impairment in color vision, and optic disc edema with splinter hemorrhage. Only patients with normal C-reactive protein levels and erythrocyte sedimentation rates were included. Eyes suspected to have arteritic anterior ischemic optic neuropathy (AAION) based on clinical and laboratory results were excluded from the study. Eyes with intraocular pressure (IOP) greater than 21mmHg, glaucomatous optic nerve damage, glaucomatous visual field defects, a history of ocular surgery (including cataract surgery), ocular trauma, corneal scars, corneal or lens abnormalities (dense cataract) that could affect corneal and anterior segment biomechanical measurements, and refractive errors exceeding ±3 diopters spherical equivalent were excluded.

**Figure 1:**
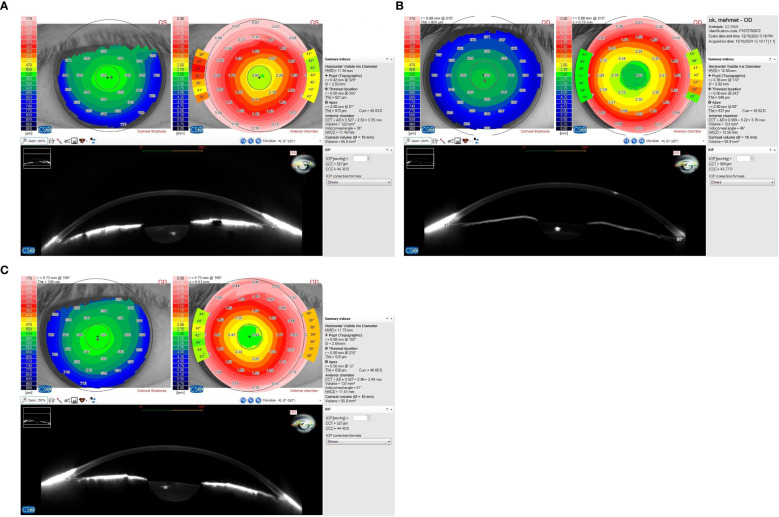
Anterior chamber topographic data of the affected **(A)** and fellow **(B)** eyes of a patient with non-arteritic anterior ischemic optic neuropathy (NAION) and an eye in the control group **(C)**.

A complete ophthalmological and neuro-ophthalmological examination was applied to all participants, including best-corrected visual acuity (BCVA) with Snellen charts, pupillary light reaction, evaluation for the presence of relative afferent pupillary defect, color vision test with Ishihara plates, slit-lamp examination, IOP measurement with a Goldmann applanation tonometer, and funduscopy. Anterior chamber depth (ACD), ACV, central corneal thickness (CCT), and iridocorneal angle (ICA) values were automatically created by the Scheimpflug/Placido photography-based topography system and a Sirius topographer (Costruzione Strumenti Oftalmici, Florence, Italy) using Phoenix v2.6 software. A single clinician (B.B.C.) took three measurements for each eye and the measurements with best alignment and fixation were included. Due to the inability to obtain reliable refraction measurements in some eyes with NAION, AL was assessed as an alternative. The ALs were measured with an IOL-Master 500 (Carl Zeiss, Meditec). Affected and fellow eyes of the patients in the study group and the right eyes of the control group were evaluated. **Figure 1** presents topographic data from the patients with NAION and from the control subjects.

All data were analyzed using SPSS v25.0 for Windows (SPSS Inc., Chicago, IL). The distribution of continuous variables was evaluated using the Shapiro–Wilks test and visual graphs (histogram, etc.). Continuous variables are presented as mean ± standard deviation. Differences between the affected eyes, fellow eyes in the study group, and the eyes in the control group were analyzed using ANOVA with Tukey’s *post hoc* tests. Statistical significance was accepted at p< 0.05.

## Results

There was no difference between the two groups in terms of age and gender (p_1_ = 0.761 and p_2_ = 0.189, respectively). **Table 1** presents the demographic characteristics of the patients in the study and control groups. When comparing the eyes with NAION, fellow eyes, and the eyes in the control group, no statistically significant differences were found in the mean values of the IOP (p=0.454). The mean AL was 22.95 ± 0.68 mm in the eyes with NAION, 22.95 ± 0.81 mm in the fellow eyes, and 23.13 ± 0.80 mm in the eyes of the control group, with no statistically significant difference among the groups (p=0.587). Since the eyes that underwent cataract surgery were excluded, all the eyes were phakic. Of the eyes with NAION, 16 were right eyes.

**Table 1:** Demographic characteristics of the patients in study (nonarteritic anterior ischemic optic neurupathy) and control (healthy) groups.

Variable	Study Group (n=28)	Control Group (n=29)	P value
Age, y, mean ± SD	58.64 ± 7.86	57.86 ± 11.19	0.761
Sex, n (%)			
Male	11(39.3)	17(58.6)	0.189
Female	17(60.7)	12(41.4)	
Hypertension (%)			
Yes	9 (32.1)	0 (0.0)	0.001*
Diabetes mellitus (%)			
Yes	11 (39.3)	0 (0.0)	0.000*
Eye (right/left)	16/12	29/0	0.000*

*Statistically significant.

There was no difference in the mean CCT between the eyes with NAION, fellow eyes, and the eyes in the control group (p=0.849). The mean ACD in the aforementioned groups was 2.64 ± 0.31mm, 2.66 ± 0.27 mm, and 2.82 ± 0.57 mm, respectively (p=0.203). The assessment of mean ACV showed that the values were 117.86 ± 22.23 mm^3^, 118.07 ± 23.34 mm^3^, and 137.93 ± 41.01mm^3^ in the eyes with NAION, fellow eyes, and eyes in the control group, respectively, demonstrating a statistically significant difference among the groups (p=0.019). *Post hoc* analyses revealed that ACV was significantly lower in the eyes with NAION and fellow eyes compared to the healthy eyes in the control group (p_1_ = 0.038, p_2_ = 0.040). There was no significant difference between the mean ICA values of the eyes with NAION, fellow eyes, and the eyes in the control group (p=0.552) (**Table 2**).

**Table 2:** Comparison of the anterior segment measurements in the affected and fellow eyes of the patients in the study group and control group.

Parameter	NAION, N=28	Fellow eyes, N=28	Control, N=29	P-value*	NAION Vs. control	Fellow eyes vs. control	NAION vs. fellow eyes
CCT, µm ± SD	544.68 ± 31.26	547.03 ± 36.93	542.48 ± 19.39	0.849	0.959	0.835	0.953
ICA, ° ± SD	40.14 ± 7.04	39.75 ± 6.40	41.62 ± 6.99	0.552	0.693	0.557	0.975
ACV, mm^3^ ± SD	117.86 ± 22.23	118.07 ± 23.34	137.93 ± 41.01	0.019	0.038	0.040	1.000
ACD, mm ± SD	2.64 ± 0.31	2.66 ± 0.27	2.82 ± 0.57	0.203	0.236	0.323	0.988
Axial length, mm ± SD	22.95 ± 0.68	22.95 ± 0.81	23.13 ± 0.80	0.587	0.651	0.640	1.000
IOP, mmHg ± SD	15.46 ± 2.65	15.57 ± 2.75	14.79 ± 2.13	0.454	0.576	0.478	0.986

*ANOVA test comparing the three groups with Tukey’s *post hoc* analysis.

ACD, anterior chamber depth; ACV, anterior chamber volume; CCT, central corneal thickness; ICA, iridocorneal angle; IOP, intraocular pressure; NAION, non-arteritic anterior ischemic optic neuropathy; SD, standard deviation.

## Discussion

The anterior segment morphology of the eyes with NAION was evaluated in this study. ACV was found to be significantly lower in the eyes with NAION and the fellow eyes compared to the healthy eyes in the control group. However, with respect to CCT, ACD, and ICA, there were no significant differences between the three groups. The findings of this study provide important insights into the anterior segment structures of eyes with NAION and their fellow eyes.

Considering that the sclera, cornea, peripapillary ring, and lamina cribrosa are composed of similar extracellular matrix components, it is plausible that their biomechanical and anatomical features may also share similarities (11). Corneal thickness and corneal hysteresis have previously been investigated in patients with NAION. Jabaly-Habib et al. hypothesized that the cornea may be thinner in patients with NAION, and that, in parallel, their crowded optic discs may also be more susceptible to damage; however, they did not find a statistically significant difference (11). In another study, CCT was found to be thinner in the affected and fellow eyes of NAION patients compared to the control eyes, although the difference did not reach statistical significance (12). In a comparative analysis of 33 NAION patients and 33 healthy individuals, Uysal et al. reported that corneal hysteresis, which represents the dynamic deformation response of the cornea, and corneal resistance factor, which reflects corneal elasticity and structural resistance, were both lower in the affected and fellow eyes of NAION patients than in control eyes (12). The authors concluded that this corneal biomechanical weakness may indirectly reflect the structural fragility of the lamina cribrosa.

To the best of our knowledge, no prior study has specifically investigated the anterior chamber characteristics of eyes with NAION. However, Cankaya et al. examined 200 healthy eyes and reported an association between anterior segment morphology and the optic nerve head (13). They also emphasized a negative correlation between rim volume and both ACD and ACV, thereby supporting the potential role of anterior segment changes accompanying a crowded optic disc in the etiopathogenesis of NAION. The development of NAION after intravitreal injection and cataract surgery has been reported, suggesting the hypothesis that anatomical variations in the anterior chamber may lead to transient IOP spikes in susceptible eyes, which may result in impaired perfusion of the optic nerve head (14, 15).

In this study, in addition to a reduced ACV, ACD was also found to be shorter in the eyes with NAION and the fellow eyes compared to the healthy controls; however, the difference did not reach the level of statistical significance. Another important finding was that the ICA did not differ significantly between the eyes with NAION and the control eyes. Although some studies reported the coexistence of NAION and angle-closure glaucoma, no comparative study has been published on the relationship between angle-closure glaucoma and NAION (16–21). In accordance with these reports, an abrupt rise in IOP with acute angle closure can trigger hypoperfusion and ischemia of the optic nerve head and the development of NAION. As far as we know, anterior chamber findings were evaluated for the first time in this study and ACV was found to be lower in both eyes of the patients with NAION, while ICA, although narrower, did not reach statistical significance.

In the present study, the prevalence of hypertension and diabetes was significantly higher in the patients with NAION compared to the control group, as expected, given that both diseases are considered risk factors for NAION (2). To our knowledge, there is no existing data in the literature demonstrating a relationship between systemic hypertension and anterior chamber parameters. However, in diabetes, lens abnormalities and reduced corneal sensitivity are frequently observed and they may indirectly influence the anterior segment (22). Accordingly, future research should be designed to include only non-diabetic NAION patients in order to eliminate the potential confounding effects of diabetes.

In light of current knowledge, the presence of a “disc at risk” appearance in the fellow eye of a patient presenting with signs of ischemic optic neuropathy is one of the most important clinical clues directing the clinician toward a non-arteritic etiology (23, 24). In this study, a reduced ACV was also identified as a potential risk factor for NAION. However, larger studies including a greater number of patients, particularly those involving eyes with AAION, which plays a critical role in the differential diagnosis of NAION, are needed to validate our findings.

The small sample size is one of the major limitations of this study, and future studies involving a larger number of patients are warranted. In addition, the absence of certain parameters, such as corneal hysteresis and lens thickness, further weakens the strength of the findings. Considering that diabetes may indirectly affect anterior chamber measurements, the inclusion of diabetic patients represents another limitation of the present study. Therefore, evaluating only non-diabetic patients with NAION in future research may provide more accurate and generalizable results.

## Conclusion

Differences in anterior segment morphology were identified in both NAION eyes and fellow eyes compared to healthy eyes. Reduced ACV may be a potential risk factor for NAION; however, larger comparative studies are needed to confirm this finding.

The authors have no funding and conflicts of interest to disclose.

The datasets generated during and/or analyzed during the current study are available from the corresponding author on reasonable request.

## Data Availability

The raw data supporting the conclusions of this article will be made available by the authors, without undue reservation.
